# A case report of a tongue ulcer presented as the first sign of occult tuberculosis

**DOI:** 10.1186/s12903-019-0764-y

**Published:** 2019-04-29

**Authors:** Seo-Yeong Kim, Jin-Seok Byun, Jae-Kap Choi, Jae-Kwang Jung

**Affiliations:** Department of Oral Medicine, School of Dentistry, Kyungpook National University, 2175 Dalgubeol-daero, Jung-gu, Daegu, 41940 South Korea

**Keywords:** Oral tuberculosis, Oral ulceration, Tongue

## Abstract

**Background:**

Tuberculosis (TB) is a serious infectious disease with considerable fatality, typically affecting the pulmonary system and, rarely, other body organs including the oral cavity. Due to the rarity of oral TB, it is frequently overlooked in differential diagnosis of oral lesions. Despite a declining trend in TB incidence in recent years, it is still a major public health problem with high contagiousness, thereby requiring the early diagnosis and prompt treatment.

**Case presentation:**

A 57-year-old male patient presented with chief complaint of painful ulcer on tip of his tongue. He reported that the ulcer developed without any remarkable event such as mechanical trauma, vesicle formation or systemic illness. His past medical history revealed the TB over 40 years ago, which had reportedly healed after pharmacological treatments. As the ulceration persisted after topical steroid application and careful education about avoiding possible mechanical stimuli, biopsy was performed and histological finding showed typical findings of oral tuberculosis including intense granulomatous inflammatory features with small red rods of mycobacterial organisms as well as epithelioid cells and Langhans giant cells. After suitable antituberculosis treatments, oral tuberculosis ulcer was almost completely healed. We present a case of oral TB affecting tip of the tongue in a patient with a history of pulmonary TB and emphasize the understanding of intraoral manifestations for early diagnosis and prompt treatment of TB.

**Conclusions:**

The present case represented the importance of understanding oral tuberculosis manifestations for dental clinicians who might be frequently the first health care professionals to encounter various oral lesions.

## Background

Tuberculosis (TB) is a chronic infectious disease usually caused by *Mycobacterium tuberculosis* (*M. tuberculosis*) that is transmitted by expelled infectious aerosol droplets of patient with active TB [1]. However, in majority of cases, those infections are suppressed by an effective immune response from the host; TB often becomes asymptomatic in latent state and is not generally contagious [2]. Approximately 5% of otherwise healthy adults will develop into active TB disease within 2 years [3].

TB is classified clinically as pulmonary and extrapulmonary, depending on the area of infection [4]. The lung is the predominant site of TB, but any organ of the body may be involved. Extrapulmonary involvement in TB is uncommon, accounting for approximately 10 to 15% of all TB patients [4]. The lymph nodes are the second most common location of TB [5]. Oral TB has been generally regarded as a rare occurrence. It is estimated that only 0.05 to 5% of total TB cases may present with oral manifestations [6]. Oral manifestations usually present as superficial ulcers, patches, papillomatous lesions, or indurated soft tissue lesions [7].

Early diagnosis and prompt treatment of TB is essential because a delay in diagnosis may have serious consequences due to the progressive and contagious nature of TB. Oral lesion can be the first manifestation of TB, even if it is rare [8]. Dentists could be the first health care professionals to recognize oral lesions of TB with unusual and abnormal progress.

A case of oral tuberculosis ulcer with poor response to topically applied steroids in a patient with latent TB is presented herein.

## Case presentation

A 57-year-old male patient visited the Department of Oral Medicine, Kyungpook National University Dental Hospital with the chief complaint of painful ulcer on the tip of the tongue. The ulcer had developed 3–4 weeks ago without any apparent initiating event such as trauma. He described a pricking sensation and an increased soreness at the tongue tip area upon touching. The patient’s medical history revealed a diagnosis of TB over 40 years ago. He reported that complete recovery was gained at that time.

**Fig. 1:**
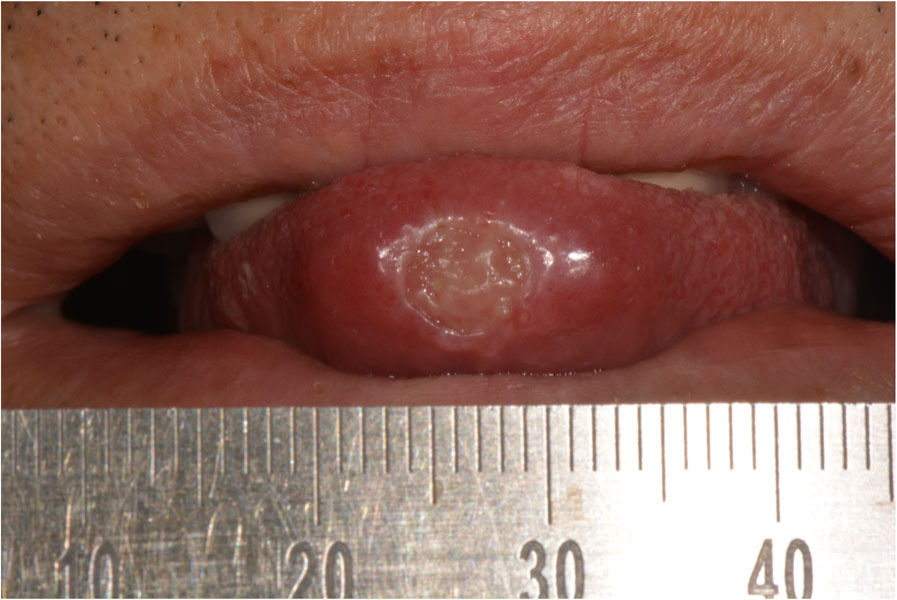
Intraoral photograph showing a round ulcer on the tip of the tongue

Intraoral examination revealed a round ulcer measuring approximately 0.7 cm in diameter on the tip of the tongue. The ulcer was characterized by a granulomatous center and a whitish, well-defined border with slight elevation (Fig. 1). The base of lesion was firm in consistency on digital palpation. Extraorally, there was no evidence of lymph node involvement. A panoramic radiograph showed no evidence of bone involvement. The laboratory examinations showed that complete blood count (CBC) was within normal limits. Serologic tests for human immunodeficiency virus and hepatitis C also revealed negative findings. Based on the clinical examination, differential diagnosis included major aphthous ulcer, traumatic ulcer, granulomatous diseases, and infections.

Topical mouthwash with a mixture of amoxicillin 1.0 g and prednisolone 30 mg in 500 mL distilled water was used for 7 weeks with careful instruction to avoid possible stimuli, and triamcinolone acetonide 5 mg was also injected into lesion twice for 2 months. Despite subtle improvement after these conservative managements, the ulcer had not completely disappeared. Biopsy was eventually performed to rule out malignancy.

**Fig. 2:**
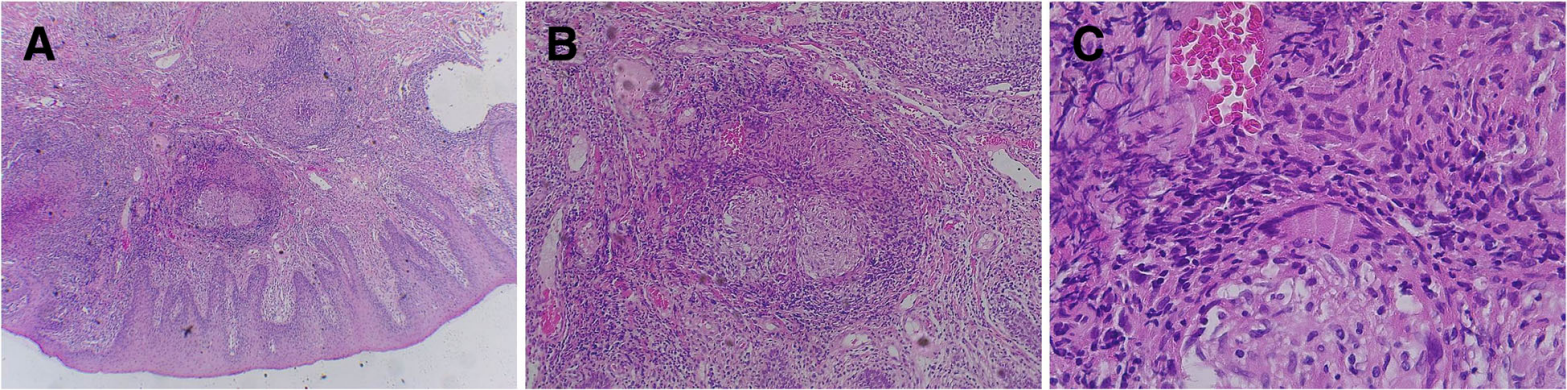
Histopathological findings. Hematoxylin-eosin staining. **a** Oral mucosa with intense granulomatous inflammation, 40x magnification. **b** Epithelioid cells and Langhans giant cells, 100x magnification. **c** The presence of an aggregate of small red rods of mycobacterial organisms, 400x magnification

An incisional biopsy of the ulcer was carried out under local anesthesia (2% lidocaine with epinephrine 1:100,000). Histological examination revealed the presence of numerous epithelioid cells and multiple Langhans giant cells and Ziehl–Neelsen staining demonstrated acid-fast bacilli (AFB) (Fig. 2). Based on histological findings, the oral ulcer was finally diagnosed as lingual TB.

**Fig. 3:**
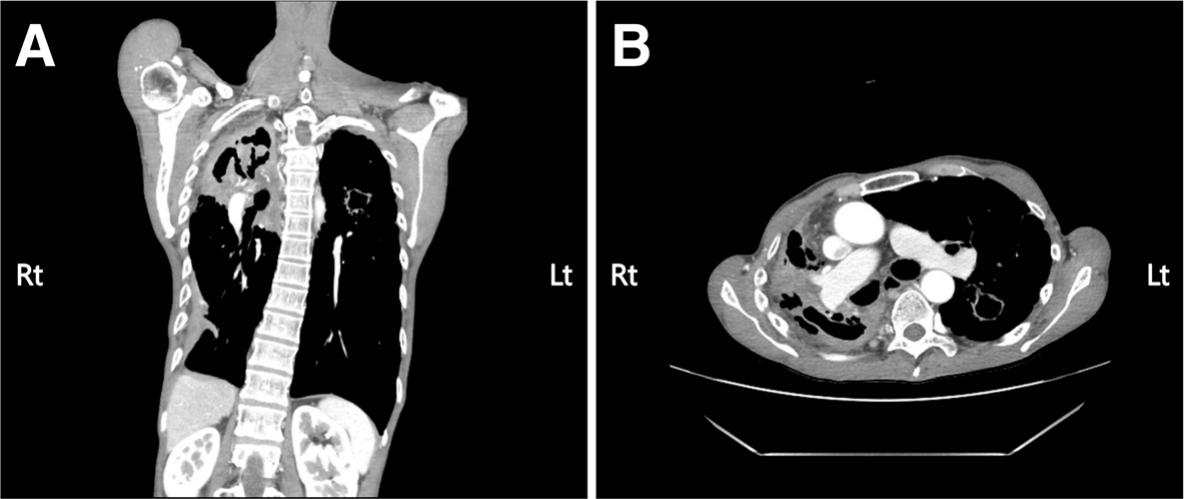
Chest computed tomography showing consolidation and fibrothorax in right lung and the cavitary lesion with clustered micronodules in left lung. **a** Coronal view. **b** Horizontal view

The patient was immediately referred to a pneumologist for further examination and management. AFB stains of lesion were positive for *M. tuberculosis*. AFB cultures were positive for *M. tuberculosis* complex. Polymerase chain reaction (PCR) was conducted on his sputum, and analysis confirmed the presence of *M. tuberculosis.*. Additional blood biochemistry revealed the increased values of erythrocyte sedimentation rate (ESR) (103 mm/h) and c-reactive protein (CRP) (2.54 mg/dL). An IFN-γ release assay (IGRA) using the QuantiFERON-TB Gold in-tube method was positive. Chest computed tomography (CT) showed destructive findings with consolidation and fibrothorax in right lung and formation of cavitary lesion with clustered centrilobular micronodules in left lung apex (Fig. 3).

**Fig. 4:**
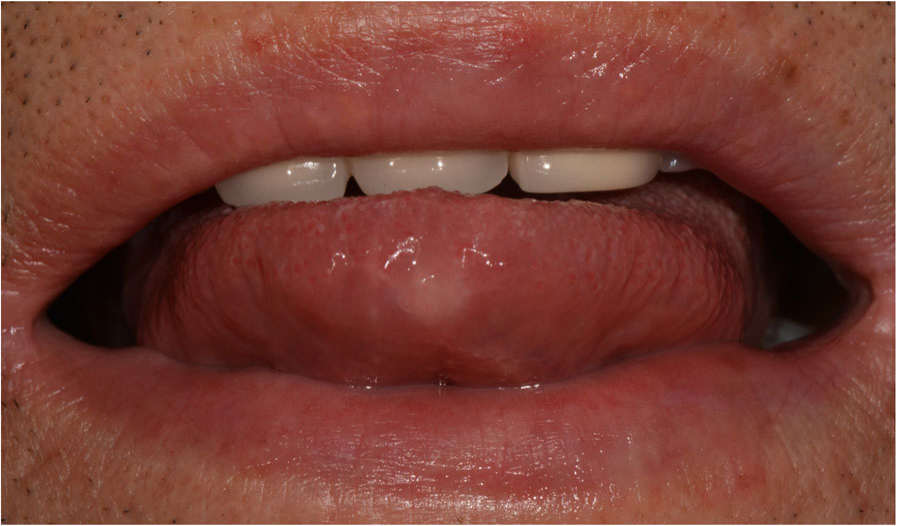
Intraoral photograph presenting the lesion almost relieved after 2 months of anti-tuberculosis therapy

**Fig. 5:**
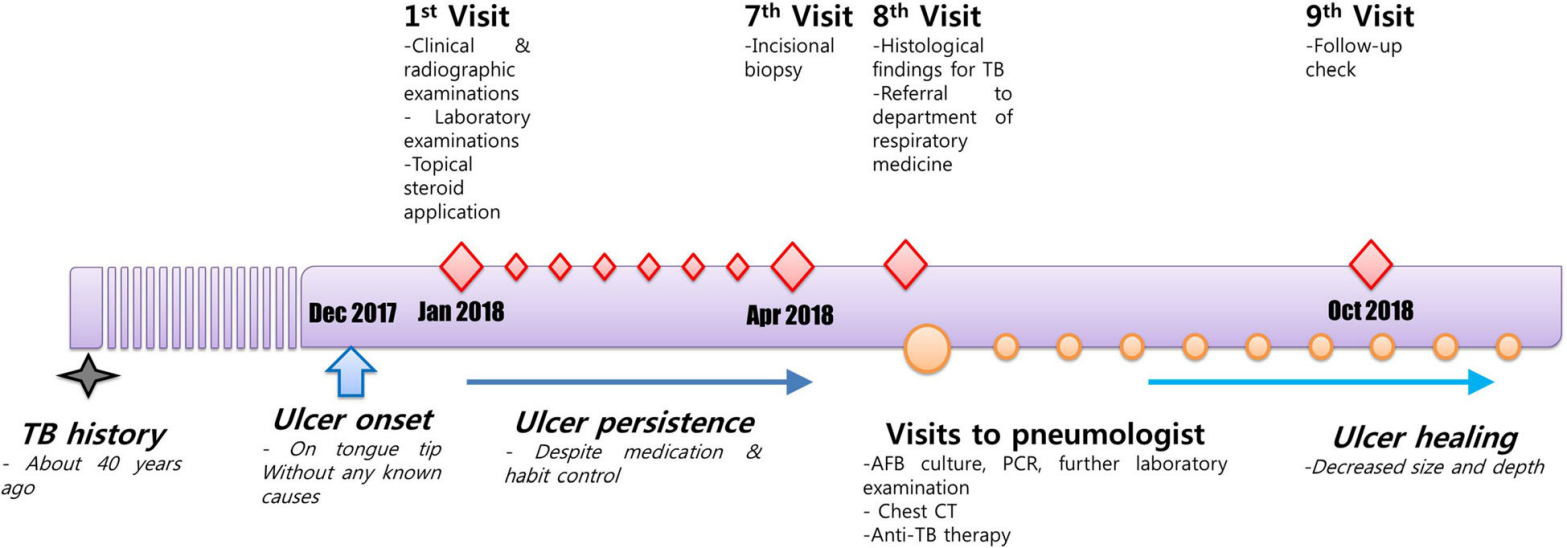
Timeline of onset and progress

After about 2 months of drug therapy, the oral ulcer of patient almost disappeared (Fig. 4), and after another 2 months, AFB culture showed no growth of *M. tuberculosis* in 4 weeks. The patient was followed up for 9 months without any complications (Fig. 5).

## Discussion

TB is the ninth leading cause of death worldwide and leading cause from a single infectious agent, ranking above HIV/AIDS. The World Health Organization estimated that about 10.4 million people were infected with TB in 2016: 90% were adults, 65% were male, 10% were people living with HIV (74% in Africa), and 56% were living in five countries, namely India, Indonesia, China, the Philippines, and Pakistan [9]. While the global incidence of TB is slowly falling by about 2% per year, it was reported that 39,000 people were still infected with TB and 2600 people died from it in 2016 in South Korea [9, 10].

The most vulnerable region to TB is pulmonary system, and infection does not usually spread to other parts of the body in most patients. However, in rare instances, progressive pulmonary TB spreads by self-inoculation via infected sputum, blood, or lymphatic system to cause secondary lesions of TB at organs other than the lung [11]. Extrapulmonary TB of pleura, lymphatics, bone, genitourinary system, meninges, peritoneum, or skin occurs in approximately 15% of TB patients [12]. TB can affect the head and neck region, including the oral cavity. Oral TB lesions may either be primary or secondary to pulmonary TB, with secondary lesions being more common. The oral lesions usually present with a stellate ulcer, most commonly on dorsum of the tongue [4].

Lingual TB may appear as ulcers, nodules, fissures, tuberculomas, or granulomas. The most frequent lesion is a superficial ulcer, characterized by undermined edges, a granulating floor, and occasional small tuberculous nodules around the periphery [13]. The ulcer may be ragged and indurated and is often painful [14]. The histological criteria for a diagnosis of oral TB include presence of granulomatous inflammation with epithelioid cells and Langhans giant cells or AFB seen on Ziehl-Neelsen staining of biopsy specimens [6].

Once in the lung, bacilli are subjected to phagocytosis and degradation by resident macrophages. However, some bacilli can escape lysosomal delivery and survive within the macrophage. *M. tuberculosis* remaining in macrophages is kept in check within the granulomas, which are clusters containing mycobacteria-infected macrophages in the center, surrounded by different types of immune cells such as macrophages, T and B lymphocytes, dendritic cells, endothelial cells, fibroblasts, and granulocytes. Mycobacteria might exist in a so-called ‘dormant’ state as long as host immunity is effective [12]. Therefore, presence of granuloma indicates the balance between host resistance and *M. tuberculosis* virulence as distinct feature of latent pulmonary TB. Many asymptomatic humans hold virulent bacteria in granulomas. While controlling the infection of bacteria, granulomas also serve as a hideout for long-term survival of bacteria [15].

Once patients are diagnosed with TB, personalized treatments should be performed with anti-TB drugs on the basis of clinical examination. Treatment of latent TB infection is also required to inhibit the development of active TB disease in those already infected with *M. tuberculosis* [9]. The oral lesions can be relieved after pharmaceutical management of systemic TB.

Oral lesions of TB exhibit a nonspecific clinical presentation and are often overlooked in differential diagnosis, even by dentists. However, when oral lesions do not adequately respond to local treatments, dentists should include TB in differential diagnosis. Careful anamnesis and clinical and radiological examination could play a leading role in clinical diagnosis of TB. In the present case, aphthous ulcer and traumatic ulcer could be ruled based on the prolonged course of the ulcer and the absence of trauma in the history. As various types of oral ulcers might also be caused by other systemic diseases—including Crohn’s disease, syphilis, blastomycosis infection, and even Langerhans cell histiocytosis, histopathological examination and culture of microorganisms should be considered for definite diagnosis [16, 17].

In our case, it was initially difficult to differentiate TB from other focal lesions because patient did not present any other systemic symptoms except for persistent oral ulcer. Considering his medical history of TB over 40 years ago, it was possible that *M. tuberculosis* TB incubated in his lung reactivated into an active and virulent state.

Recently, despite the decreasing tendency of developing new cases of TB in Korea, TB incidence and mortality rate in Korea are still the highest among Organization for Economic Co-operation and Development (OECD) countries. As dentists are the first health care professionals that frequently encounter various oral lesions, it is important to understand the various oral manifestations of TB for avoiding a delayed diagnosis and poor prognosis. In summary, oral TB should be included in differential diagnosis of persistent oral lesions when diagnosing patients with a history of TB, even though evidence of TB is rare in oral cavity. Accurate diagnosis is critical for optimal treatment by focusing on the pathological source, to avoid inappropriate oral therapy.

## Abbreviations

AFB: Acid-fast bacilli
CBC: Complete blood count
CRP: C-reactive protein
CT: Computed tomography
ESR: Erythrocyte sedimentation rate
IGRA: IFN-γ release assay
M. tuberculosis: *Mycobacterium tuberculosis*
OECD: Organization for Economic Co-operation and Development
PCR: Polymerase chain reaction; TB: tuberculosis

## References

[1] Cole EC, Cook CE (1998). Characterization of infectious aerosols in health care facilities: an aid to effective engineering controls and preventive strategies. Am J Infect Control.

[2] Persing DH (2015). Latent tuberculosis: interferon and beyond?. J Mol Diagn.

[3] Menzies NA, Wolf E, Connors D, Bellerose M, Sbarra AN, Cohen T (2018). Progression from latent infection to active disease in dynamic tuberculosis transmission models: a systematic review of the validity of modelling assumptions. Lancet Infect Dis.

[4] Aoun N, El-Hajj G, El Toum S (2015). Oral ulcer: an uncommon site in primary tuberculosis. Aust Dent J.

[5] Ju W, Fu Y, Liu Y, Tan Y, Dong M, Wang L (2018). Clinical and pathologic analyses of tuberculosis in the oral cavity: report of 11 cases. Oral Surg Oral Med Oral Pathol Oral Radiol Endod..

[6] Wang W, Chen J, Chen Y, Lin L (2009). Tuberculosis of the head and neck: a review of 20 cases. Oral Surg Oral Med Oral Pathol Oral Radiol Endod..

[7] Miziara ID (2005). Tuberculosis affecting the oral cavity in Brazilian HIV-infected patients. Oral Surg Oral Med Oral Pathol Oral Radiol Endod.

[8] Krawiecka E, Szponar E (2015). Tuberculosis of the oral cavity: an uncommon but still a live issue. Postepy Dermatol Alergol.

[9] World Health Organization. Global tuberculosis report 2017: World Health Organization; 2017. Available from: https://www.who.int/tb/publications/global_report/gtbr2017_main_text.pdf.

[10] World Health Organization. Global tuberculosis report 2013: World Health Organization; 2013. Available from: https://apps.who.int/iris/bitstream/handle/10665/91355/9789241564656_eng.pdf.

[11] Rivera H, Correa M, Castillo-Castillo S, Nikitakis N (2003). Primary oral tuberculosis: a report of a case diagnosed by polymerase chain reaction. Oral Dis.

[12] Dube D, Agrawal GP, Vyas SP (2012). Tuberculosis: from molecular pathogenesis to effective drug carrier design. Drug Discov Today.

[13] Pande TK, Hiran S, Rao V, Pani S, Vishwanathan K (1995). Primary lingual tuberculosis caused by M. bovis infection. Oral Surg Oral Med Oral Pathol Oral Radiol Endod..

[14] Von Arx D, Husain A (2001). Oral medicine: Oral tuberculosis. Br Dent J.

[15] Gupta A, Kaul A, Tsolaki AG, Kishore U, Bhakta S (2012). Mycobacterium tuberculosis: immune evasion, latency and reactivation. Immunobiology..

[16] Facciolo MT, Riva F, Gallenzi P, Patini R, Gaglioti D (2017). A rare case of oral multisystem Langerhans cell histiocytosis. J Clin Exp Dent.

[17] Mortazavi H, Safi Y, Baharvand M, Rahmani S (2016). Diagnostic features of common Oral ulcerative lesions: an updated decision tree. Int J Dent.

